# Excited State Dynamics in Unidirectional Photochemical Molecular Motors

**DOI:** 10.1021/jacs.4c01019

**Published:** 2024-04-24

**Authors:** Palas Roy, Andy S. Sardjan, Wesley R. Browne, Ben L. Feringa, Stephen R. Meech

**Affiliations:** † School of Chemistry, University of East Anglia, Norwich NR4 7TJ, U.K.; ‡ Centre for Systems Chemistry, Stratingh Institute for Chemistry, University of Groningen, 9747AG Groningen, The Netherlands; § Molecular Inorganic Chemistry, Stratingh Institute for Chemistry, University of Groningen, 9747AG Groningen, The Netherlands; ∥ School of Basic Sciences, Indian Institute of Technology Bhubaneswar, Bhubaneswar, Odisha 752050, India

## Abstract

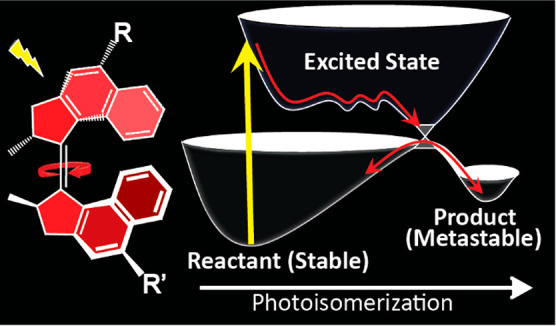

Unidirectional photochemically driven molecular motors (PMMs) convert the energy of absorbed light into continuous rotational motion. As such they are key components in the design of molecular machines. The prototypical and most widely employed class of PMMs is the overcrowded alkenes, where rotational motion is driven by successive photoisomerization and thermal helix inversion steps. The efficiency of such PMMs depends upon the speed of rotation, determined by the rate of ground state thermal helix inversion, and the quantum yield of photoisomerization, which is dependent on the excited state energy landscape. The former has been optimized by synthetic modification across three generations of overcrowded alkene PMMs. These improvements have often been at the expense of photoisomerization yield, where there remains room for improvement. In this perspective we review the application of ultrafast spectroscopy to characterize the excited state dynamics in PMMs. These measurements lead to a general mechanism for all generations of PMMs, involving subpicosecond decay of a Franck–Condon excited state to populate a dark excited state which decays within picoseconds via conical intersections with the electronic ground state. The model is discussed in the context of excited state dynamics calculations. Studies of PMM photochemical dynamics as a function of solvent suggest exploitation of intramolecular charge transfer and solvent polarity as a route to controlling photoisomerization yield. A test of these ideas for a first generation motor reveals a high degree of solvent control over isomerization yield. These results suggest a pathway to fine control over the performance of future PMMs.

## 1 Introduction

Molecular machines deliver directional molecular motion at the nanoscale in response to external stimuli.^1−11^ The conversion of energy into useful directional motion, mechanical work, is familiar from macroscopic machines. However, molecular machines must do their work under the perpetual bombardment of Brownian motion, so the work done takes on a more explicitly statistical character.^12−15^ Consequently, the operation and function of molecular machines are different from those of their macroscopic analogues, as has been discussed in detail elsewhere.^13,16^ The two major stimuli available to drive molecular machines are light and chemical energy.^17^ Nature makes use of each in the “electromechanical” machinery of vision and the mechanical motion of kinesin, for example. These two fundamentally different stimuli give rise to a further distinction in the mechanism of molecular machines, in part because absorption of a photon (*hν* ≫ *kT*) allows population of states that are not accessible at or near thermal equilibrium.^12,18,19^ In this perspective the focus is on the group of light activated molecular machines that exhibit intramolecular rotational motion: the photochemical molecular motors (PMMs). Specifically, we are concerned with PMMs in which directional molecular motion arises from excited state isomerization reactions.^20^

**Figure 1:**
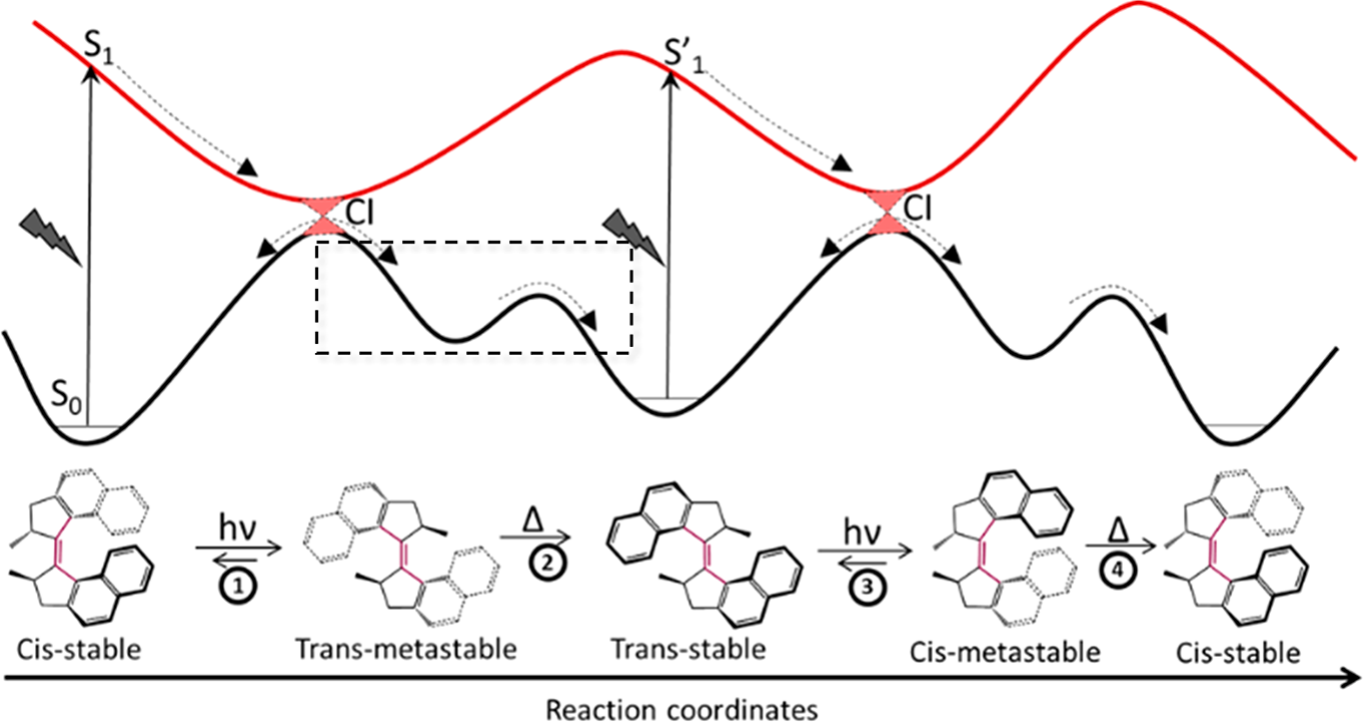
General mechanism for the first generation PMM **1**. The molecular structures and corresponding PESs are shown for the four-step cycle involving two photoisomerizations (*hν*) and two THI steps (Δ); the boxed step is analyzed in detail by ultrafast spectroscopy later.

The archetypal PMM is the overcrowded alkene dimethyl tetrahydro-bi(cyclopenta[α]naphthalenylidene) **1** (Figure 1).^21,22^ The stilbene motif is readily apparent at the heart of this structure, and stilbene is known to undergo a reversible light driven cis–trans isomerization.^23,24^ That and similar reactions (in azobenzenes, for example^25^) have found many applications in light activated switches and sensors, but they do not have the ability to support the repeated *unidirectional* motion characteristic of a motor.^8,17,26^ The directionality in **1** comes about initially from steric crowding. Specifically, in the trans stable form there is steric repulsion between the methyl groups at each of the two stereo centers and the naphthalene moieties. This interaction twists the rings out of plane, giving **1** a helical character and giving rise to exciton coupling between the aryl rings. The helicity is manifested in their circular dichroism spectra.^22,27^ Consequently, upon electronic excitation of a ππ* transition localized on the ethylenic bond, which reduces its bond order, the initial rotation will be overwhelmingly in the energetically favorable direction determined by steric repulsion. This is the first step in the rotation about the double bond “axle”. It is worth mentioning here two points of nomenclature.^14^ Reference to an “axle” is certainly appropriate (Figure 1), but it should be recalled that, unlike a propeller, the molecule has 3*N* – 6 modes of motion several of which are involved in PMM function, in addition to the obvious axle torsion.^28−30^ Further, the primary light driven step in PMMs has been referred to by us and others as the “power stroke”. The nomenclature of motor steps has been a topic of discussion relating to the mechanical/statistical nature of the underlying processes.^12,14^ “Power stroke” was used in analogy to the Otto cycle of the four-stroke motor but is not exact; “ignition” may be equally appropriate (though not “combustion”!). Even that analogy is unsatisfactory as the PMM requires two ignitions for one rotation: here we will use the more literal “absorption”. The net result of absorption and the initial photochemical reaction is the cis product in the electronic ground state, where the rotation about the double bond has caused a change in configuration, such that the methyl groups adopt a sterically strained pseudoequatorial orientation. This metastable state is of higher energy than the initial trans ground state but cannot relax back to it, due to the high energy barrier associated with rotation of the re-formed double bond (Figure 1). However, this metastable ground state can relax in the forward rotation direction to populate a stable cis configuration via a thermal helix inversion (THI), allowing the methyl groups to adopt the energetically favorable pseudoaxial orientation; at this point the motor has completed one-half rotation. This stable cis product is shown in Figure 1, from which it is apparent that THI indeed requires significant structural reorganization. There is thus an energy barrier associated with this relaxation and the THI is the rate-determining step in PMM rotation. Further, the stable cis form is structurally different from the initial trans form, is of lower energy, and has a distinct electronic spectrum. Absorption of a second photon is required to complete the rotation. Again, absorption reduces bond order on the axle allowing low barrier or barrierless rotation on the excited state potential energy surface (PES), which again occurs in the forward direction of rotation by virtue of steric repulsion. The reaction leads to the trans electronic ground state, with the methyl groups now adopting the unfavorable equatorial orientation (Figure 1). This metastable trans form relaxes back to the initial stable trans structure by rotating in the forward direction via a second THI, completing one full rotation. The general scheme in Figure 1 was proven by observing the four different states which have distinct electronic and NMR spectra. Directionality was further demonstrated by chiroptical spectroscopy associated with successive THI reactions.^21^

Since the THI is the rate-determining step, the frequency of PMM rotation can be optimized by careful synthetic modification of energy barriers in the electronic ground state (Figure 1).^7^ Tremendous progress has been made in this area, and megahertz rotation frequencies can in principle be achieved. Of course at two photons per rotation and for a significant populations of PMMs the experimental design must pay attention to light intensity and heat dissipation, and the fastest motors are photokinetically limited.^1,31−34^

The efficiency of any PMM is a product of the frequency of rotation and the quantum yield of the excited state isomerization reaction.^35^ A low yield for photoisomerization means that most absorbing molecules repopulate the original ground state and the absorbed energy is wasted as heat. It is thus important to optimize both yield and frequency (but these are not independent—see below). Photoisomerization yield optimization presents specific challenges, not least because excited state PESs are less well characterized and more difficult to calculate than the ground state surfaces. Further, the photochemical yield will be determined by the nature of the coupling between ground and excited states, i.e., the position and topology of conical intersections (CIs).^36−39^ How these are modified by chemical substitution and changes in the environment must be understood if photochemical yield is to be optimized to match the advances in PMM frequency already achieved.

The photochemical yield is clearly controlled by events in the excited electronic state, so excited state dynamics are critical. In this perspective we describe recent ultrafast time resolved studies of three generations of overcrowded alkene based PMMs, which lead us to a general mechanism for their underlying excited state dynamics. These are complemented by descriptions of quantum chemical calculations made by others, which have proven essential in interpreting the observed dynamics in terms of underlying nuclear motions. Based on this general model the effects of solvent and different substituents can be rationalized and exploited in the design of PMMs with optimal yields. Finally, this work on overcrowded alkene motors will be placed in the context of other families of PMMs that rely on light driven double bond isomerization reactions. The work ends with some conclusions and prospects for further work and finally a description of the main experimental methods.

## 2 Excited State Dynamics in Second Generation PMMs

**Figure 2:**
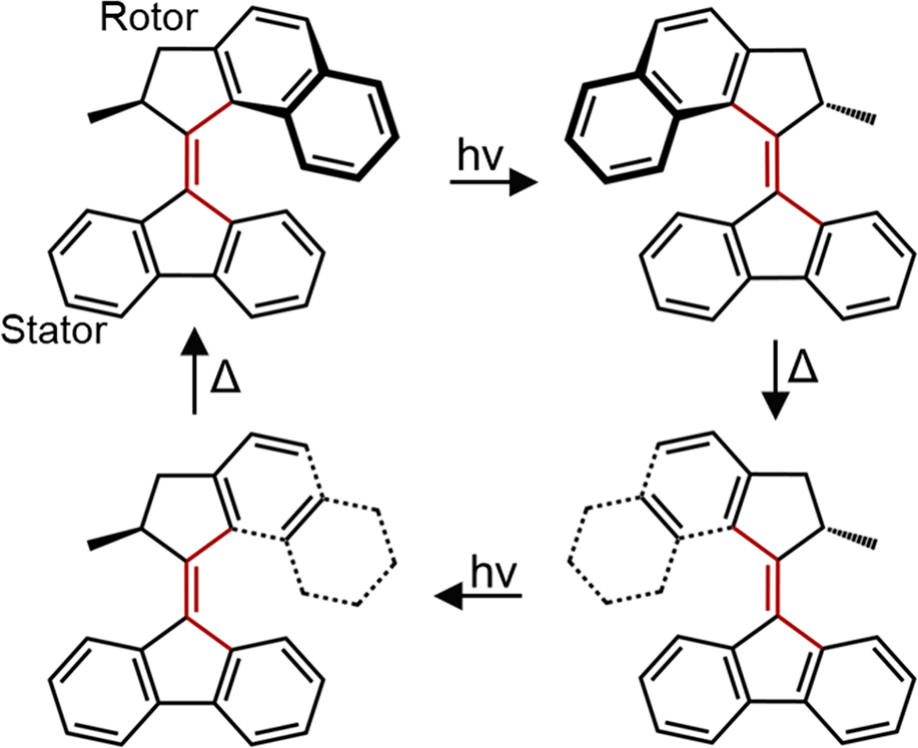
General mechanism for operation of a second generation motor, here of the fluorene stator design, **2**, showing successive photochemical and thermal steps. Adapted from ref (50). Copyright 2017 American Chemical Society.

The first generation motor **1** demonstrated the proof of principle and mechanism of PMM function.^21,26^ Quantitative study of its photochemical behavior revealed a high quantum yield for photoisomerizaton, as expected for the fundamental stilbene architecture coupled to strong steric repulsion; yields in the region of 60–85% were reported.^40,41^ However, the barrier to THI in **1** is high, such that at room temperature the maximum operational frequency is restricted to a few hertz, yielding an efficiency that is too low for many applications. This limitation stimulated a great deal of synthetic effort and resulted in a family of motors with the general structure **2** (Figure 2) comprising a larger aromatic ring system called a “stator” (typically based on a fluorene, **2**, or tricyclic aromatic) linked by a double bond axle to a smaller “rotor”.^32,33,42^ The rotor typically contains a single chiral center to provide the stereochemistry required for unidirectional motion, while the stator can be modified, allowing it to be linked to other molecules or to surfaces, as the application requires.^43,44^ By modifying the nuclear and electronic structures of rotor and stator, both stereochemistry and steric crowding can be manipulated to control the barrier to THI, such that these second generation motors are capable of megahertz rotational frequencies.^32^ It is convenient to begin our discussion of PMM excited state dynamics with **2** rather than with **1** since this structure is the most widely used and has been studied in most detail by both ultrafast spectroscopy and quantum mechanical and molecular dynamics calculations.

Manipulating the barrier to enhance the rate of THI also modifies excited state dynamics and was observed to markedly reduce the quantum yield for isomerization to typically <10%.^30^ Thus, although the PMM frequency increased, the efficiency was not optimized, a fact which motivated us to investigate the excited state dynamics in **2**.^45^ As can be seen in Figure 2, the photocycle is analogous to that of **1** with successive unidirectional isomerization and THI steps.^46^ When the stator is symmetric about the axle axis, then the two stable states are identical, which simplifies study.

The steady state spectroscopy of **2** and similar motors is characterized by a strongly allowed unstructured absorption spectrum (extinction coefficients of the order of 2 × 10^4^ M^–1^ cm^–1^) in the UV (350–400 nm) region of the spectrum.^46^ The fluorescence is structureless, broad, red-shifted, and extremely weak, with a quantum yield of <10^–4^.^47^ Irradiation in the UV at low temperature leads to a new absorption band in the visible region, which reverts to the original state on warming; these spectral changes are assigned to photochemical population and thermal decay of the metastable form (Figure 2).^32,46^

### 2.1 Ultrafast Spectroscopy of **2**

**Figure 3:**
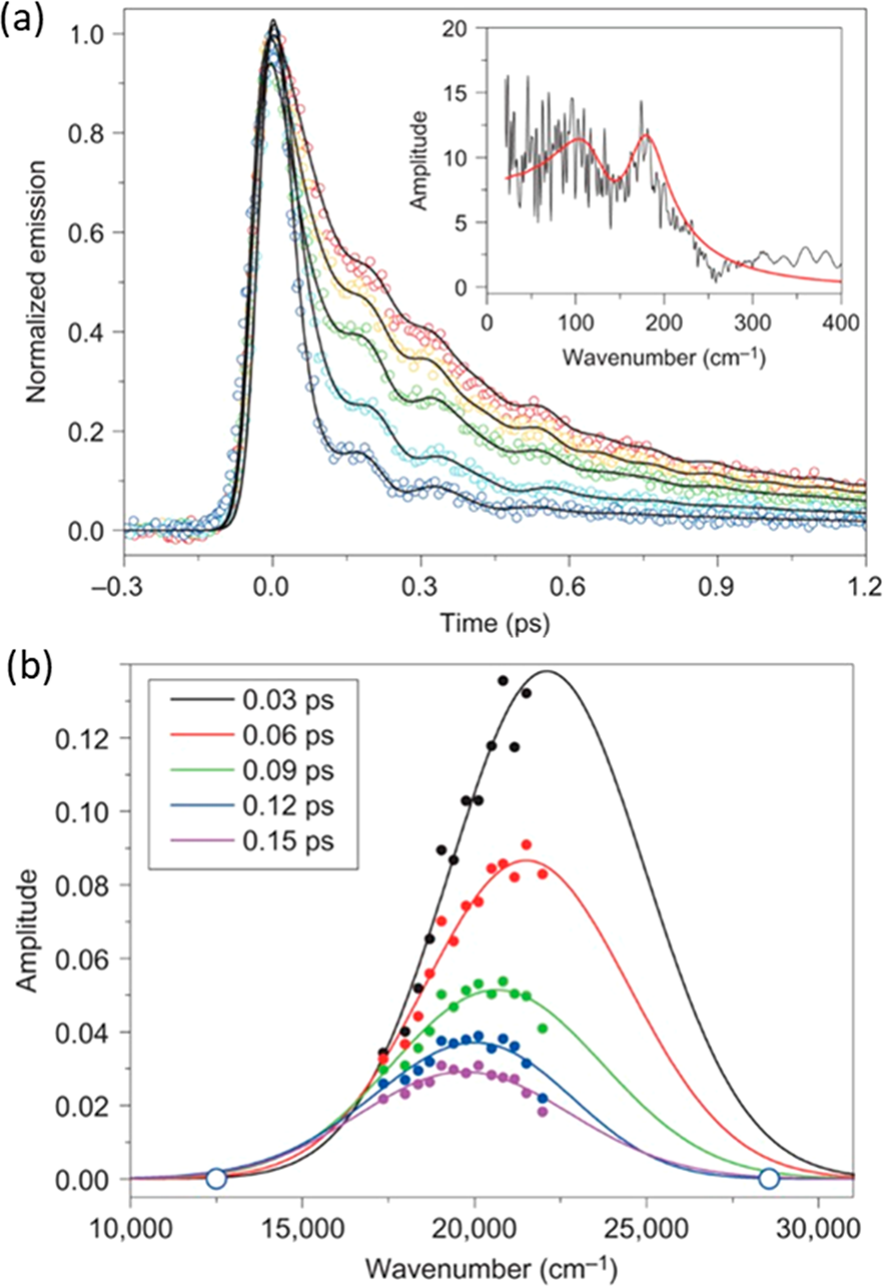
Time resolved fluorescence of **2**. (a) Wavelength resolved nonsingle exponential emission showing the blue-shifted contribution of the FC state and the coherences. The inset shows the Fourier transform of the residuals. (b) Time resolved emission spectra reconstructed from the data in (a). Adapted with permission from ref (47). Copyright 2012 Springer Nature.

Fluorescence upconversion (FlUC) measurements of the stable to metastable photochemical reaction (Figure 2) of **2** reveal two remarkable features (Figure 3a).^47^ First, the decay of the FC state is extremely fast, with a time constant of ca. 100 fs; second, the decay is nonsingle exponential, with the ultrafast FC state decay followed by a much slower component of the order of 1–2 ps. Superimposed on the fluorescence decay are clear low frequency oscillations arising from coherent excitation of low frequency modes in the excited state. These can be fit directly in the time domain or isolated by subtraction of the underlying exponential decay and analyzed by Fourier transform, with both approaches resolving two modes of 113 and 180 cm^–1^.

The fluorescence decay of **2** was monitored as a function of wavelength (Figure 3a), where it is apparent that the ca. 100 fs decay dominates on the blue edge of the emission, while the longer-lived component is of increasing weight on the red side. These data can be converted to time resolved emission spectra to reveal a rapid spectral shift to the red with time, accompanied by fast amplitude decay (Figure 3b).^47^ This is interpreted as indicating ultrafast (ca. 100 fs) relaxation out of the optically allowed (or “bright”) Franck–Condon (FC) state driven by structural relaxation due to decreased bond order and strong steric repulsion. The observation that fluorescence persists after this relaxation suggests that the PMM remains in its excited electronic state but with a reduced transition dipole moment, a “dark” state. The dark state emission is red-shifted, consistent with relaxation on the excited state PES. (It is worthwhile to add a note on nomenclature—bright and dark “states” here indicate different structures on the same lower excited state surface with distinct photophysical properties and do not necessarily indicate a change of electronic state.)

The oscillations observed arise from impulsively excited low frequency modes, i.e., modes excited by a pulse of light shorter in duration than the inverse of the frequency of the vibrational mode, such that a wave packet is launched in the excited state. These oscillations have dephasing times of >100 fs, and at least the higher frequency mode survives in the dark state. Of course the dark state is not directly excited by the pulse, but as discussed by Joo and co-workers, ultrafast structural relaxation can take the place of a short pulse and impulsively excite low frequency modes in the final state; thus, it is likely the higher frequency mode observed is excited via the ultrafast FC state decay.^48^ In contrast the lower frequency mode damps on the time scale of FC state decay and may be involved in that reaction coordinate.^47^ Significantly, these modes are observed to oscillate in phase across the entire emission spectrum, suggesting a mode which modulates the transition dipole moment rather than the energy gap between the emissive and ground states; such a coordinate dependent transition moment is called a non-Condon effect and has been reported in other isomerization reactions.^49^

The transient absorption (TA) data are consistent with this picture. Two positive ΔOD features are seen at 730 and 670 nm.^50^ The former exhibits a partial decay in the first 100 fs (at the time resolution limit of the TA) to generate the shorter wavelength absorbing transient, which has a corresponding rise time. Subsequently both states decay with a 1.6 ps time constant, and the same oscillatory features as were found in FlUC are observed. This is consistent with the mechanism described above, in which a bright excited state relaxes to a dark excited state which then decays in 1.6 ps.

Structural information on the dark excited state is accessible from femtosecond stimulated Raman spectroscopy (FSRS) measurements described in section 8 (currently the 150 fs time resolution is inadequate to resolve the bright state FSRS of **2**; see below for such measurements on the third generation PMM). The Raman pump wavelength was set to be resonant with the dark state, and the resulting excited state resonance Raman spectrum was compared with calculated and measured ground state data.^50^ Ground state Raman spectra reveal an intense pair of bands near 1600 cm^–1^ associated with the axle and ring C=C stretches, which are expected to contribute to resonance enhanced Raman, as they are displaced upon ππ* excitation. The dark state FSRS displays a single broad mode at the same wavenumber but also has major contributions at lower wavenumbers, between 1300 and 1500 cm^–1^. Ground state density functional theory (DFT) calculations suggest that C=C ring modes contribute to this region but at slightly higher wavenumbers than observed. It was suggested that the observed downshift arises from weakened bonds in the excited state following ππ* excitation.^50^

A second interesting feature of the FSRS measurements is that they reveal the complete PMM^51^ photocycle, in that formation of the metastable ground state product is resolved at long time delays (>20 ps), where the excited state has fully decayed. The resulting preresonance Raman spectrum, which is observed because the red-shifted metastable state is closer to resonance with the Raman pump wavelength, has red-shifted C=C modes compared to the stable state, in good agreement with DFT calculations.^50^

### 2.2 Solvent Effects

Solvent effects on excited state dynamics of **2** were measured. Solvents play a key role in determining the rate of excited state isomerization reactions, and they provide an important means of probing the reaction coordinate.^45^ Motion along a reaction coordinate in which a significant volume of solvent is displaced is expected to become slower in more viscous solvents. This friction effect has been investigated in detail for a number of isomerization reactions. In general the reaction rate constant can be expressed as^52^
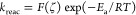
1in which *F*(ζ) is a friction dependent term that can often be modeled by a Kramer’s expression^53^ and *E*_a_ is the energy barrier along the reaction coordinate. It is common to treat friction as the solvent shear viscosity, but for many isomerization reactions this is unsuccessful, probably because macroscopic viscosity is often a poor representation of microscopic friction. The problem has been widely discussed.^51,54^ One often successful representation is to fit the measured rate constants to a power of the viscosity, *F*(ζ) ∝ η^α^, where α represents a reaction coordinate and solvent dependent constant between 0 and 1,^55,56^ with the latter indicating essentially hydrodynamic diffusive motion.^54^ Solvent polarity also plays a key role, particularly when the reaction coordinate involves a change in the extent of intramolecular charge separation. In that case the charge separated configuration may be stabilized by polar solvent reorganization, which can modify both the pre-exponential and *E*_a_, as reviewed elsewhere.^51,57^ This interplay of barrier and friction have been studied in some detail for **1** and is discussed further in section 3.

For **2** the initial ultrafast decay is independent of solvent viscosity.^45^ This suggests that relaxation out of the FC state is driven by the strongly energetically downhill steric repulsion which dominates over any solvent friction effects. In contrast, the slower relaxation associated with dark state decay is sensitive to viscosity, indicating the involvement of diffusive molecular motion along the reaction coordinate. Ultimately, the reaction coordinate leading to isomerization must involve rotation of the rotor with respect to the stator. Rotation of such large molecular units through the solvent is indeed expected to be sensitive to solvent friction. However, the observed viscosity effect has a relatively low value of α < 0.5, possibly indicating that the reaction coordinate follows a low volume route to formation of the metastable state. In contrast to viscosity, solvent polarity had a negligible effect on the FlUC data, suggesting that charge transfer configurations do not play a major role in the excited state decay of the dark state of **2**.

### 2.3 Quantum Chemical Calculations

Further details on the nature of the reaction coordinate leading to the product isomer requires calculations of the excited state PES and its CIs with the ground state. For molecules the size of **2**, accurate calculations are computationally demanding. Further, theoretical simulation of lifetime and quantum yield data requires calculation of classical or quantum dynamics trajectories on these PESs, adding an additional layer of difficulty.

The early calculations of Kazaryan et al. on a truncated form of **2** are informative.^58,59^ They describe an excited state PES connecting the stable and metastable states comprising two important coordinates: the expected axle torsion and pyramidalization at the axle carbon atoms. Stable and metastable forms in the ground state are separated by a high potential barrier. Population of the FC state by photoexcitation of the stable form places the molecule on a steeply downhill region of the excited state PES, which leads to a shallow minimum with an axle torsion angle near 90°. Different minima were accessed depending on whether the initial state was the stable or metastable form. Two CIs with the ground state were identified, both involving pyramidalization at carbon atoms of the ethylenic axle. Of these, the CI with pyramidalization at the fluorene ring was readily accessible from the excited state minimum, while the other was at higher energy. Thus, the reaction pathway was dominated by this CI. This picture was largely confirmed by Li and Morokuma using higher level methods.^60^

Kazaryan et al. studied excited state dynamics through semiclassical trajectory surface hopping calculations.^58^ Indeed, they confirmed that the majority of reactive pathways involved excited state relaxation to the ground state, with relaxation occurring close to the energetically accessible CI. Calculation of sufficient numbers of trajectories allows determination of the mean excited state lifetime and (by continuing the calculation on the ground state PES) the quantum yield. The calculated mean lifetime for the truncated **2** was in remarkably good agreement with the measured 1.6 ps dark state lifetime of **2**. However, some population was trapped for longer times in the excited state minimum, and the calculated quantum yield for isomerization was higher than observed (ca. 0.4 compared to a measured value of 0.14 for **2**(45)).

Kazaryan et al. also calculated the excited state lifetime following initial population of the FC state of the metastable form and determined it to be slightly longer than for the stable form.^58^ This difference points to a complex multiminima landscape of the PES in the dark state. There are fewer experimental studies of excited state dynamics in metastable states, but Hall et al. prepared and selectively excited the metastable state of **2**.^61^ They found that the dark state lifetime was indeed different compared to stable state excitation, consistent with the complex landscape of the PES minimum calculated. However, the experimental result revealed a shorter lifetime for the metastable state, in disagreement with the calculations. It is likely that a more detailed comparison of calculation and measurement will require inclusion of the effects of the solvent environment on the PES and reaction pathway.

**Figure 4:**
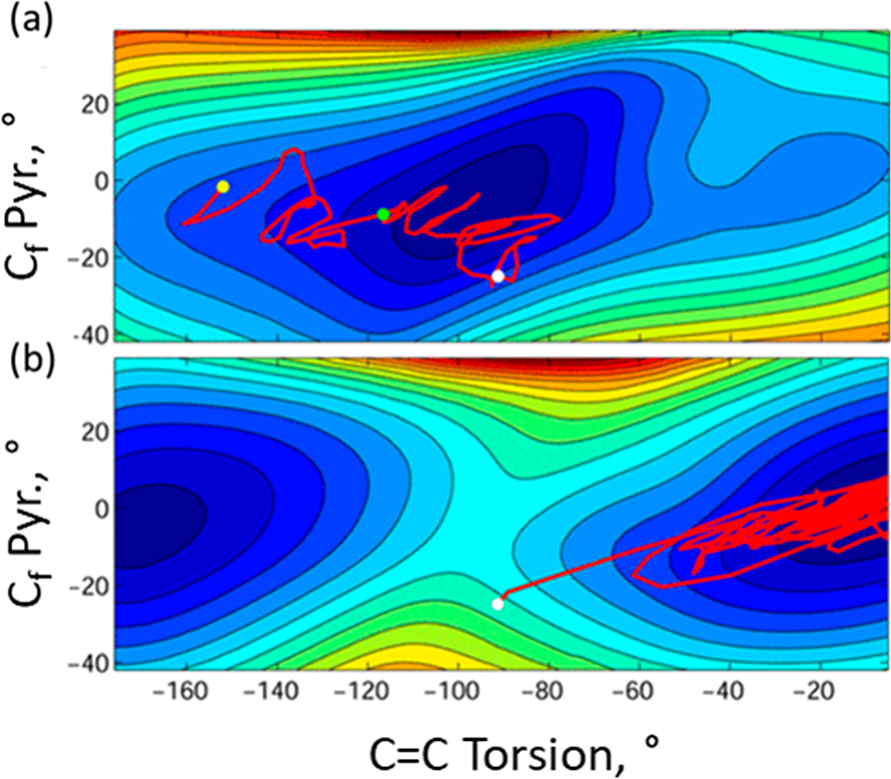
Example of a trajectory calculation. (a) The excited state dynamics on the torsion–pyramidalization surface: yellow circle = FC state, green = onset of the dark state, and white = CI. (b) The subsequent formation of the product state. Adapted from ref (62). Copyright 2017 American Chemical Society.

Further progress was reported by Pang et al., who made trajectory calculations on the metastable state of **2**.^62^ The PES and CIs calculated were largely in agreement with earlier studies, with the key coordinates again being axle torsion and pyramidalization. An example of a trajectory calculated by Pang et al. leading to the product state is shown in Figure 4. The key additional data in these calculations was the explicit incorporation of the time resolved fluorescence through determination of its transition dipole moment. The evolution of the transition moment for emission revealed an energetically downhill ca. 200 fs evolution from a bright FC state to a dark state, which was followed by picosecond time scale access to the CI, through which the ground state is populated. This is in good agreement with experiment (Figure 3).^47^ Further, the modest 30° torsion involved in reaching the dark state is consistent with the solvent viscosity independent ultrafast relaxation (Figure 4). Recently the population dynamics of **2** were calculated including the effect of solvent. Again, the agreement with experiment was good, including the modest polarity effect on dark state lifetime.^63^

### 2.4 Substituent Effect

Following from the characterization of excited state dynamics in **2**, a key objective is to use those results as a basis for rational synthetic modification to optimize the photochemical quantum yield. Clearly the photochemically active region is the axle, so electron withdrawing and donating substituents were placed on the rotor in conjugation with the central double bond, but located so as not to increase the volume of the isomerizing group (and hence modify viscosity effects).^45,64^ Three derivatives were studied. The photochemical quantum yield was measured and revealed a significant substituent effect, with electron withdrawing and donating groups, respectively, promoting and suppressing product isomer formation.^45^ FlUC and TA also showed significant substituent effects. The ultrafast bright to dark state transition was observed in all cases, but the transition dipole moment associated with the dark state showed a strong substituent dependence, as did its lifetime. Specifically, the more electron withdrawing the substituent, the higher the isomerization quantum yield, the lower the dark state transition moment, and the longer its lifetime.

**Figure 5:**
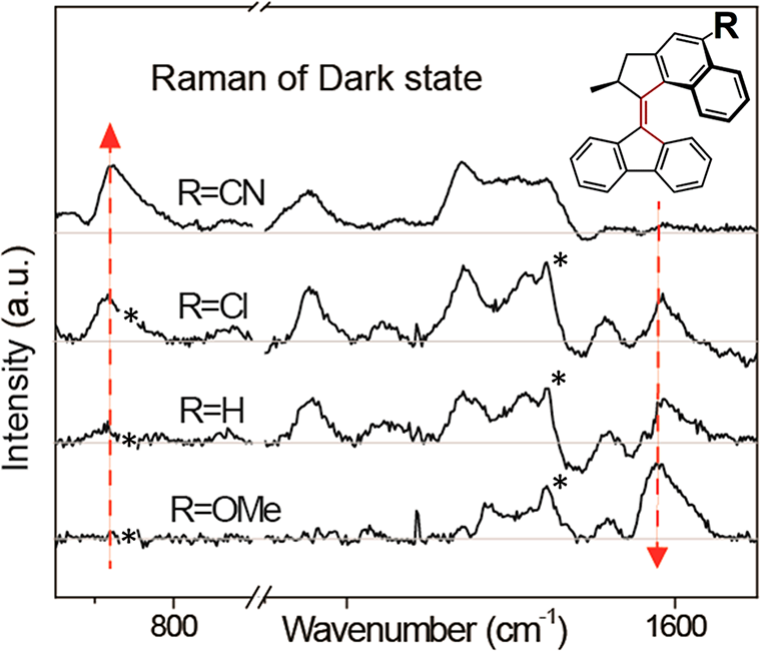
Femtosecond stimulated Raman spectra of the dark excited state of molecular motor substituted forms of **2** measured at 200 fs. Asterisks represent solvent and instrumental artifacts. Red dashed arrows represent regions where substituent dependence is greatest. Adapted from ref (64). Copyright 2021 American Chemical Society.

**Figure 6:**
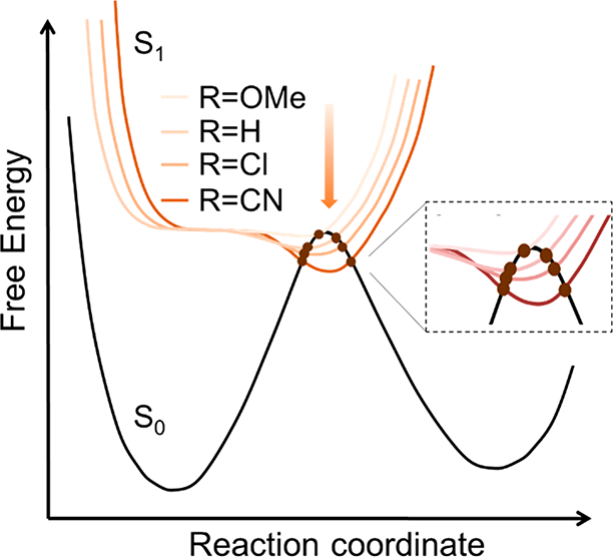
A schematic one-dimensional representation of how electron withdrawing substituents enhance the yield while extending the dark state decay time of **2**. Brown disks represent CIs, transmission through which will favor either the product or the original ground state, depending on substituent. The barrier between the dark state minimum and CI (largest for CN, negligible for OMe) controls the dark state lifetime. Adapted from ref (45). Copyright 2014 American Chemical Society.

FSRS showed that these substituents influence dark state structure (Figure 5).^64^ While electron donating substituents favored the band assigned to C=C stretches near 1600 cm^–1^ in the excited state, this mode was essentially absent for the cyano substituent, which instead revealed a new mode in the dark state near 750 cm^–1^. In resonance Raman spectra, modes which gain intensity are those which are displaced between ground and resonant excited states. Based on this and calculations of the ground state Raman spectrum, the low frequency mode was tentatively assigned to a pyramidalization mode, in line with quantum chemical calculations of the reaction coordinate. The changes in dark state structure and lifetime certainly correlate with the higher isomerization quantum yield, but understanding whether that reflects changes in the PES or reengineering the topology of the CI to favor the product channel awaits further calculations. A schematic representation of substituent effects on the PES is shown in Figure 6; this model was developed further in studies of first generation motors.

## 3 Controlling Photochemical Yield in First Generation Motors

### 3.1 Ultrafast Spectroscopy

The cis stable to trans metastable photochemical conversion of **1** (Figure 1) was studied by FlUC.^40^ Qualitatively the behavior is similar to **2**, an ultrafast (ca. 100 fs) decay on the blue edge of the emission relaxing to a red-shifted excited state with a much lower transition dipole moment—the dark state. The dark state decay reveals oscillations consistent with coherent excitation of low frequency modes that modulate the transition dipole moment. The principal differences with **2** are that the dark state of **1** is less dark (the emission has greater amplitude) and longer lived (extending to tens of picoseconds).

The longer dark state lifetime of **1** allows its decay to be probed in more detail. The picosecond dark state decay kinetics of **1** were observed to be nonsingle exponential but with only a modest wavelength dependence.^40^ The nonsingle exponential decay is consistent with the PES in the region of the dark state having multiple minima and pathways to the CI. As already indicated for **2**, it is expected that the isomerization dynamics are to some extent dependent on solvent viscosity (eq 1). Increasing the viscosity of the solvent indeed led to a significant increase in the mean dark state lifetime of **1**, consistent with an isomerization reaction that displaces solvent volume during dark state relaxation. The decay time was observed to scale as approximately the square root of viscosity.

The longer dark state lifetime of **1** suggests a barrier in the isomerization coordinate. Extraction of the barrier from an Arrhenius analysis is challenging, as the decay is viscosity dependent (eq 1), and viscosity is also a function of temperature. The barrier can be isolated by an “isoviscosity analysis” where a series of similar solvents are studied at temperatures where their viscosities are the same. An Arrhenius analysis applied to these isoviscosities yields the activation energy. This analysis was conducted for the cis to trans photoisomerization of **1** in a series of alkane solvents, revealing a low activation barrier of 3.4 kJ mol^–1^ for dark state decay.^65^ However, when the measurements were repeated in a series of linear primary alcohols a negative activation energy was recovered, suggesting a factor other than viscosity and barrier height is important in the decay pathway. Further, a comparison of polar and nonpolar liquids of similar viscosities showed that polar solvents enhance dark state radiationless decay, independent of the viscosity. Since the series of *n*-alcohols have different—and temperature dependent—polarities, this polarity effect will perturb the isoviscosity analysis, leading to the anomalous activation energy observed.

The observation of a polarity effect on the cis–trans photoisomerization in **1** provides further insight into the reaction coordinate. The steady state absorption and emission spectra are only weakly dependent on solvent polarity, suggesting that the polarity dependence arises late in the reaction coordinate, after the dark state has formed. The origin of this effect can be understood from earlier experimental and theoretical studies of photoisomerization in ethylenic compounds; indeed, a similar solvent polarity effect was noted for the stilbene isomerization and assigned to solvent stabilization of a polar transition state.^52^ Such a polar transition state arises in (nearly) symmetric ethylenic molecules such as **1** due to the *sudden polarization* as the axle CC bond torsion angle approaches 90°.^66,67^ At this point an oscillating charge difference exists between the two carbon centers of the overall nonpolar bond. In ethylene in the gas phase the charge may be localized by pyramidalization at the C atom, yielding a polarized bond. In the solution phase the symmetry may also be broken by the solvent polarization, transiently favoring charge localization.^68^ In either case the transient polarization of the bond that results may be further stabilized by polar solvent reorganization. The joint operation of axle torsion and pyramidalization at the fluorene carbon was already described for **2** above and treated in detail in the calculations of Kazaryan et al.^58,59^ The same arguments are expected to apply equally to the isomerization of **1**, with solvent dynamics and pyramidalization combining to stabilize the transition state, reducing the barrier in the pathway to the CI in polar solvents. In general, the result indicates that the excited state dynamics calculations in PMMs should include not only the coupled torsion–pyramidalization nuclear motion but also the polar solvent dynamics as they respond to stabilize charge separation at the axle.

Thus, a common picture for the dynamics of **1** and **2** emerges from ultrafast experiments and the corresponding calculations. Initial excitation of the bright FC state is followed by fast (ca. 100 fs) relaxation driven by steric repulsion on the excited state torsion–pyramidalization surface. This leads to a dark excited state which relaxes to the ground state via a CI. At least in the case of **1** this occurs over a solvent dependent barrier. The coherent oscillations observed in the fluorescence transition moment can be described as oscillations in the excited state connecting the bright state to the dark state region of the PES (Figure 4). In **1** these have a well-defined frequency of 138 cm^–1^.^40^ The nonexponential decay of the dark state suggests that some of the population is transiently trapped in the dark state for tens of picoseconds, consistent with multiple minima on the PES and multiple pathways to the CI(s).

A key question is whether the observed solvent polarity induced acceleration in dark state decay also influences the isomerization quantum yield. Yield measurements in solvents of the same viscosity but different polarities showed that it does not.^65^ Evidently the pathway to the CI and the fate of the molecule after passage through it in **1** are distinct from those in **2**. However, the evidence that the polarity of the transition state can be used to modify the reactive PES prompted further studies of the effect of electron donating/withdrawing substituents; consideration of the results of some earlier quantum chemical calculations leads to a similar conclusion.^69^

### 3.2 Substituent Effects

**Figure 7:**
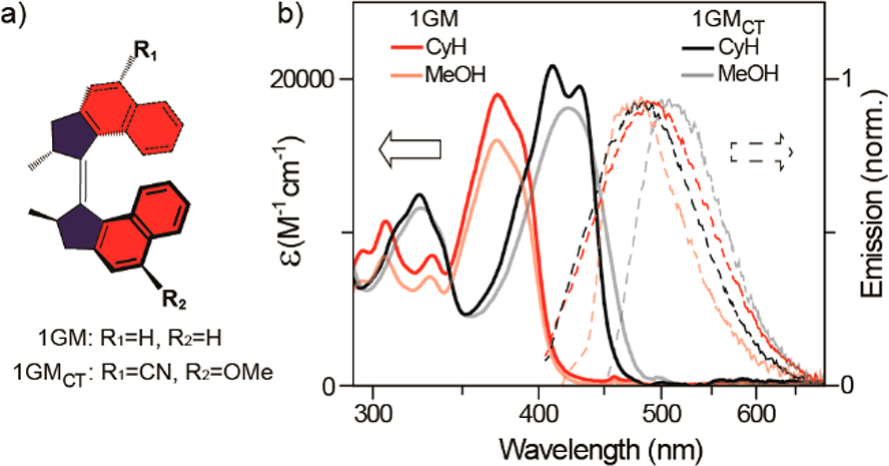
(a) Chemical structures of **1** and **1**_**CNOMe**_. The ethylenic bond is in the plane of the page, and lighter/darker bonds indicate orientation below/above the page. (b) Absorption (solid lines) and emission (dashed lines) as a function of solvent for **1** (red) or **1**_**CNOMe**_ (black). Adapted from ref (70). Copyright 2023 American Chemical Society.

**1** was modified by locating electron withdrawing (nitrile, CN) or donating (methoxy, OMe) substituents symmetrically or asymmetrically but in conjugation with the axle double bond (Figure 7a).^70^ All substituents caused a red shift in absorbance relative to **1** with CN substituents having the larger effect and the asymmetric **1**_**CNOMe**_ the largest of all (Figure 7b). There are only modest changes in the extinction coefficient and band shape, suggesting that the same ππ* electronic transition is excited in each compound. Photoisomerization quantum yields were measured for all derivatives in moderately polar CDCl_3_ using in situ NMR methods. The high quantum yield (>60%) of **1** was maintained for all samples with the exception of **1**_**CNOMe**_, which had only a 4% yield, suggesting a significant effect when substituents are positioned to favor charge separation in the excited state. Given the expected sensitivity of such charge transfer (CT) states to solvent polarity (see above), the quantum yield measurements for **1**_**CNOMe**_ and **1** were repeated in (nonpolar) deuterated cyclohexane and (polar) methanol-*d*_4_. Remarkably **1**_**CNOMe**_ had a quantum yield of 0.99 in C_6_D_12_, essentially the highest yield possible, in contrast to the (essentially polarity independent) mean value for **1** of 64%. In contrast, **1**_**CNOMe**_ in polar methanol-*d*_4_ again had a low yield of only 6%. Thus, for **1**_**CNOMe**_ the yield is enhanced to near unity in nonpolar solvents but also exhibits a remarkable sensitivity to solvent polarity not seen in other derivatives.

**Figure 8:**
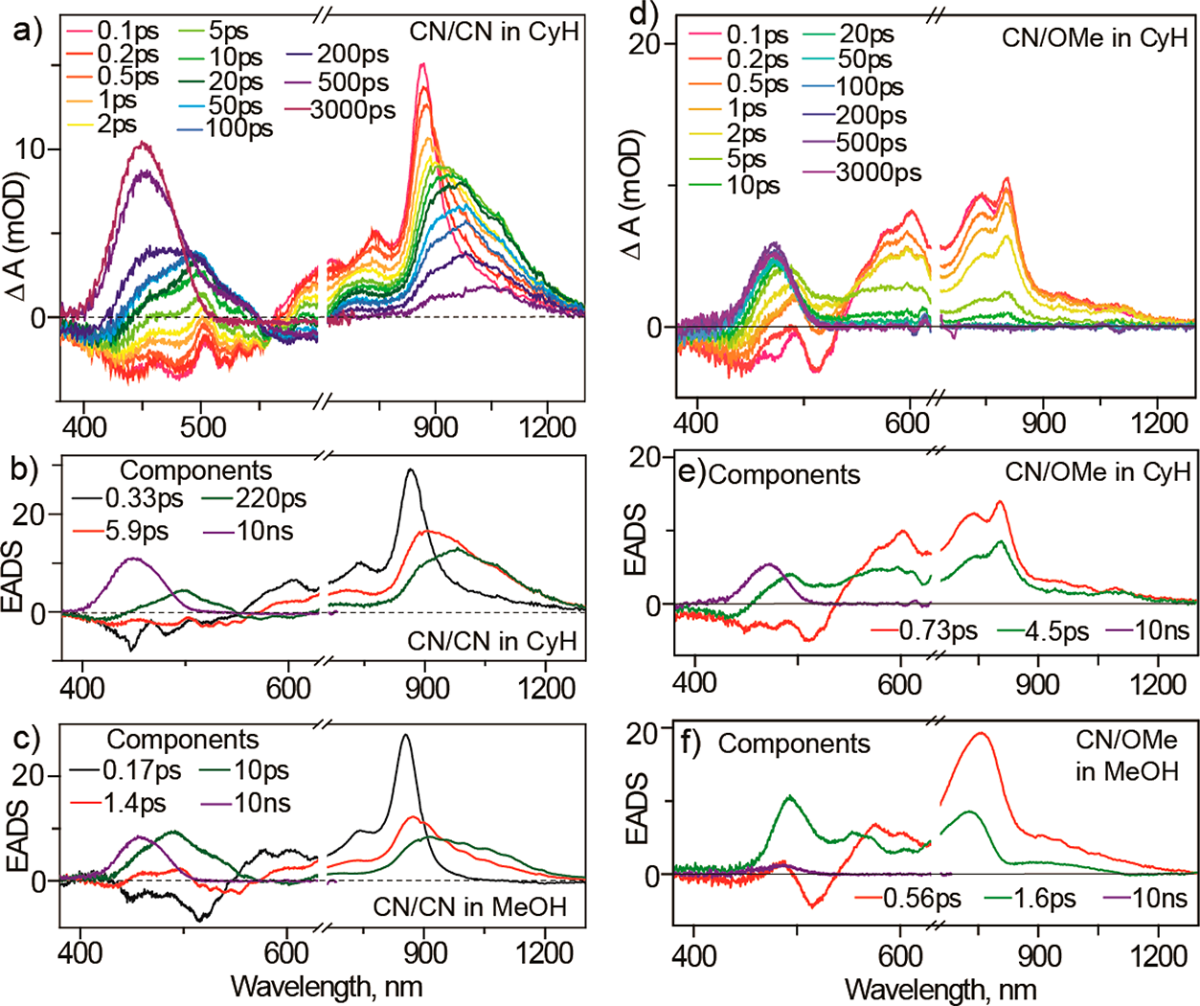
Transient absorption for first generation substituents. (a) Ultrafast evolution of TA for **1**_**CNCN**_ in cyclohexane. (b, c) EADS of **1**_**CNCN**_ in cyclohexane and methanol. (d) Ultrafast evolution of TA in **1**_**CNOMe**_ cyclohexane. (e, f) EADS of **1**_**CNOMe**_ in cyclohexane and methanol.

The same series was interrogated by FlUC, TA, and FSRS.^70,71^ The TA data are shown for **1**_**CNCN**_ in cyclohexane with the corresponding global analysis in both cyclohexane and methanol shown in Figure 8b,c. These are typical for the symmetrically substituted derivatives. The FC state has an intense absorption in the near IR and shows stimulated emission near 500 nm. In all symmetric derivatives the FC state decays in 240 ± 70 fs, independent of solvent. The dark excited state formed has absorbance shifted further into the near IR and a new absorption near 500 nm. That state exhibits a substituent and solvent dependent nonsingle exponential decay on the tens of picoseconds time scale to yield the metastable ground state product, which absorbs around 450 nm. The dynamics are accelerated in methanol, but the evolution associated difference spectra (EADS) are broadly the same. The exception to this pattern is again the “push–pull” substituted **1**_**CNOMe**_ (Figure 8d–f). In that case the FC state decay is not resolved, perhaps decaying too fast for TA or merging into the dark state, so only a nonsingle exponential decay is seen with no rising component. The faster decay component also exhibits stimulated emission while the slower one does not, suggesting that the evolution to an excited state with a lower transition moment is still a feature of the reaction coordinate. These kinetics are again accelerated in methanol, but the major difference is in the yield of the metastable product after 1 ns, apparent in the TA near 400 nm, which is ca. 10-fold higher for nonpolar than polar solvents, consistent with the photochemical quantum yield data. Thus, the polarization in the excited state due to asymmetric substitution has substantially modified the excited state dynamics and led to a remarkable solvent polarity dependence of motor efficiency.

The exceptional behavior of **1**_**CNOMe**_ is also apparent in the FlUC data, where the amplitude of the ultrafast to picosecond components (a measure of the FC state to dark state transition moment) is much larger than for any of the other derivatives, and the decay is wavelength independent.^70,71^ Further the oscillation in the FlUC data, corresponding to low frequency Raman active modes, which are observed with a variable pattern for all of the symmetric derivatives, are essentially absent for **1**_**CNOMe**_, suggesting that its CT excited state configuration has substantially reorganized the excited state PES.

In terms of controlling the efficiency of PMMs, these results are very encouraging. Studies of the excited state dynamics of first and second generation PMMs revealed a common two-step excited state decay that was well reproduced by theory. An observed solvent polarity effect suggested the imposition by substituents of CT configurations, which was realized. Such substituents both enhance the yield of the metastable product in nonpolar solvents and introduce solvent polarity control of PMM efficiency.^70^ Before summarizing PMM excited state dynamics, we will describe an extension of these ultrafast methods to the third generation of PMMs.

## 4 Excited State Dynamics in Third Generation Motors

**Figure 9:**
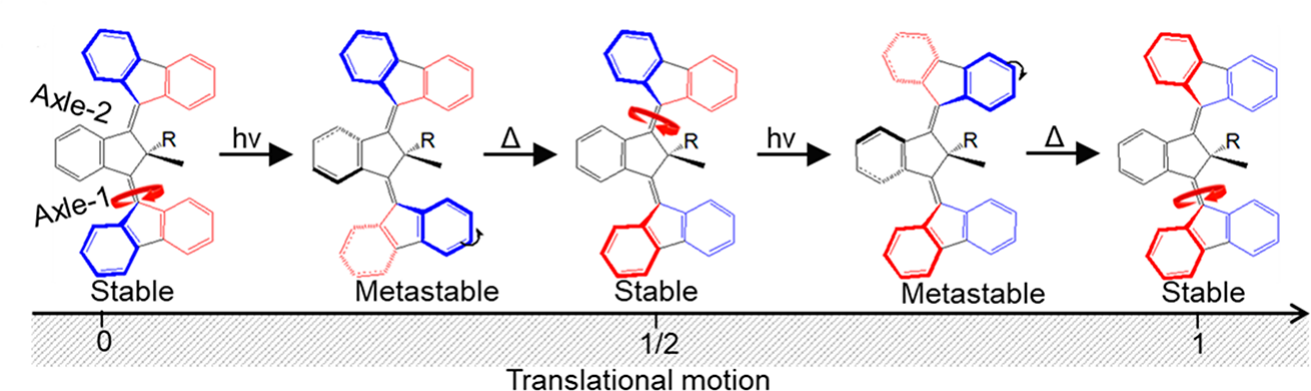
Representation of the structure of **3** and the illustration of its ability to convert motor rotation into translational motion. Adapted with permission from ref (76). Copyright 2023 Springer Nature.

At a macroscopic level the mechanical conversion of rotational to translational motion is literally prehistoric. The more recent extension to the molecular scale was achieved with an elegant synthesis linking four second generation motors at the corners of an organic framework—the “nanocar”.^72^ A third generation of PMMs has been designed to achieve rotational to translational motion more efficiently (**3**, Figure 9).^73,74^ Essentially, third generation motors are a coupled pair of second generation motors combined with opposite helicities. The required unidirectional rotations are provided by steric crowding and a single common pseudochiral center. The axle comprises a pair of ethylenic linkages, and crucially the two rotors mirror each other, so they rotate in the same direction. This molecule therefore has the potential to support translational motion, as illustrated in Figure 9. Indeed, in an elegant surface microscopy experiment this potential was recently realized with electron impact excitation of the C=C bonds.^75^

**Figure 10:**
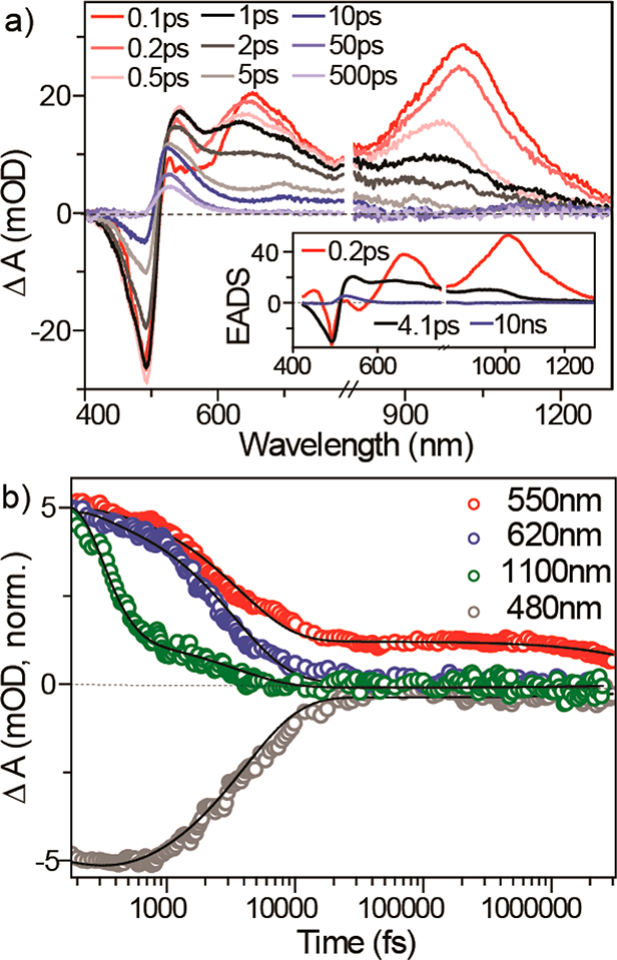
(a) Transient absorption spectra of **3** and (b) wavelength resolved amplitude of the transient kinetics fit to the FC → dark state→ product kinetics. Adapted with permission from ref (76). Copyright 2023 Springer Nature.

**3** was interrogated by ultrafast spectroscopy.^76^ Perhaps unsurprisingly, its excited state dynamics are similar to those of **2**; in FlUC experiments a blue-shifted (high energy) FC state was observed to relax in ca. 200 fs to a dark state, which supports coherently excited low frequency modes. Interestingly the low frequency modes in the excited state of **3** are observed to be sensitive to solvent polarity below 100 cm^–1^.^76^ In TA, the FC to dark state to metastable product evolution is well resolved and occurs on the few to tens of picoseconds time scale. A particularly attractive feature of the TA data for **3** is that all three states are well resolved, so their populations can be followed in real time, providing the most detailed possible insight into the PMM mechanism (Figure 10a). In particular, the ground state bleach of the stable form is well resolved and does not recover significantly during the decay of the FC state, showing that most of the excited molecules reach the dark state (Figure 10b). This is significant because in other excited state isomerization reactions a bifurcation in relaxation from the FC state has been observed, which would degrade overall efficiency.^77−79^ The absence of this effect in PMMs arises from the strong direction out of the FC state provided by steric repulsion. The dark state decay is again quenched in polar solvents, and this solvent effect is related to the yield of the final product, which forms in tandem with dark state decay. The solvent dependence may indicate a degree of charge separation in the dark state decay pathway of **3**, which correlates with the solvent dependent low frequency Raman spectrum.

It was noted that the mean dark state lifetime from the FlUC data is persistently shorter than that recovered from TA.^76^ This difference likely reflects a distribution of structures in the dark state with a distribution of radiative transition moments. The discrepancy can then be understood if the longer lifetime is associated with the structures with lower transition moments, such that measurements of fluorescence favor the faster decaying “less dark” population, while all states will contribute to the TA. Thus, this result (along with the overall nonsingle exponential decay) is further evidence for a complex PES landscape in the dark state.

**Figure 11:**
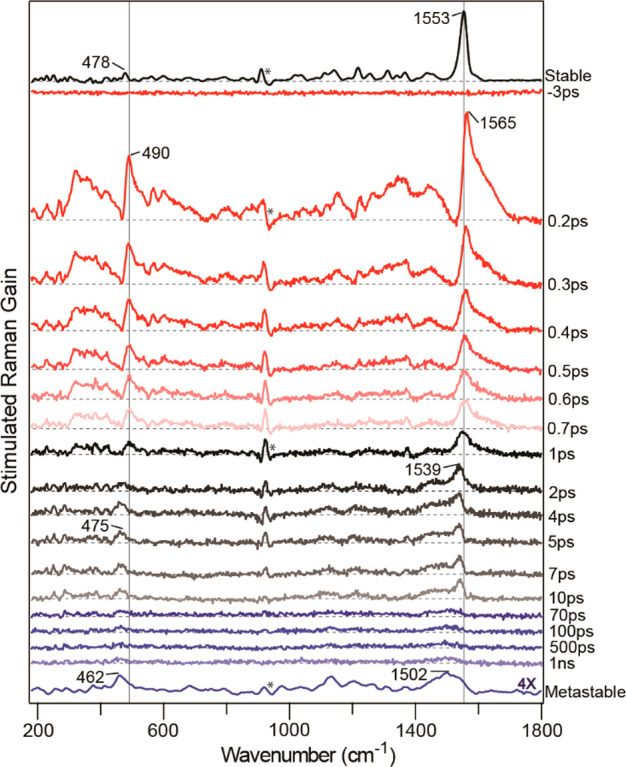
Evolution of the FSRS of **3** as the transformation of FC to dark state to product proceeds.

The structural evolution of **3** can be followed through its photocycle by FSRS (Figure 11). Excitation of the FC state yields a relatively simple Raman spectrum with a pair of modes near 1560 cm^–1^ and an enhanced (compared to the ground state Raman) mode at 490 cm^–1^. The pair of modes at higher wavenumber are associated with the C=C stretches, consistent with the observed and calculated pair at 1553 cm^–1^ in the ground state Raman. Unexpectedly, both the axle C=C modes of the FC state are observed at higher wavenumbers than in the ground state. The most blue-shifted mode is assigned to the axle C=C remaining after the initial excitation of one rotor. Initially, in the ground state the two C=C modes are coupled, but when one is excited and its bond order reduced, the other will be shifted to a higher wavenumber. The origin of the second mode is less certain but is likely to arise from a C=C ring mode perturbed upon electronic excitation. In the first few hundred femtoseconds, as the FC structure relaxes to the dark state, the higher wavenumber mode shifts to the red, merging with the lower wavenumber one. Once the dark state has formed, there is a further red shift of both modes, which eventually yields a broad Raman band at 1502 cm^–1^, the wavenumber expected for the pair of C=C modes observed and calculated for the metastable product state. Over the same time range the 490 cm^–1^ mode also red shifts, again eventually aligning with the metastable state Raman spectrum, and hence structural evolution in the entire PMM photocycle of **3** has been resolved.

Thus, excited state dynamics in the third generation motor follow closely the mechanism already characterized for first and second generation motors, but is resolved in finer detail. It would be interesting to investigate the effect of substituents on these PMMs, which in this case would be expected to support the symmetry breaking which leads to rotation of one of the two C=C bonds.

## 5 General Mechanism for Excited State Dynamics in PMMs

**Figure 12:**
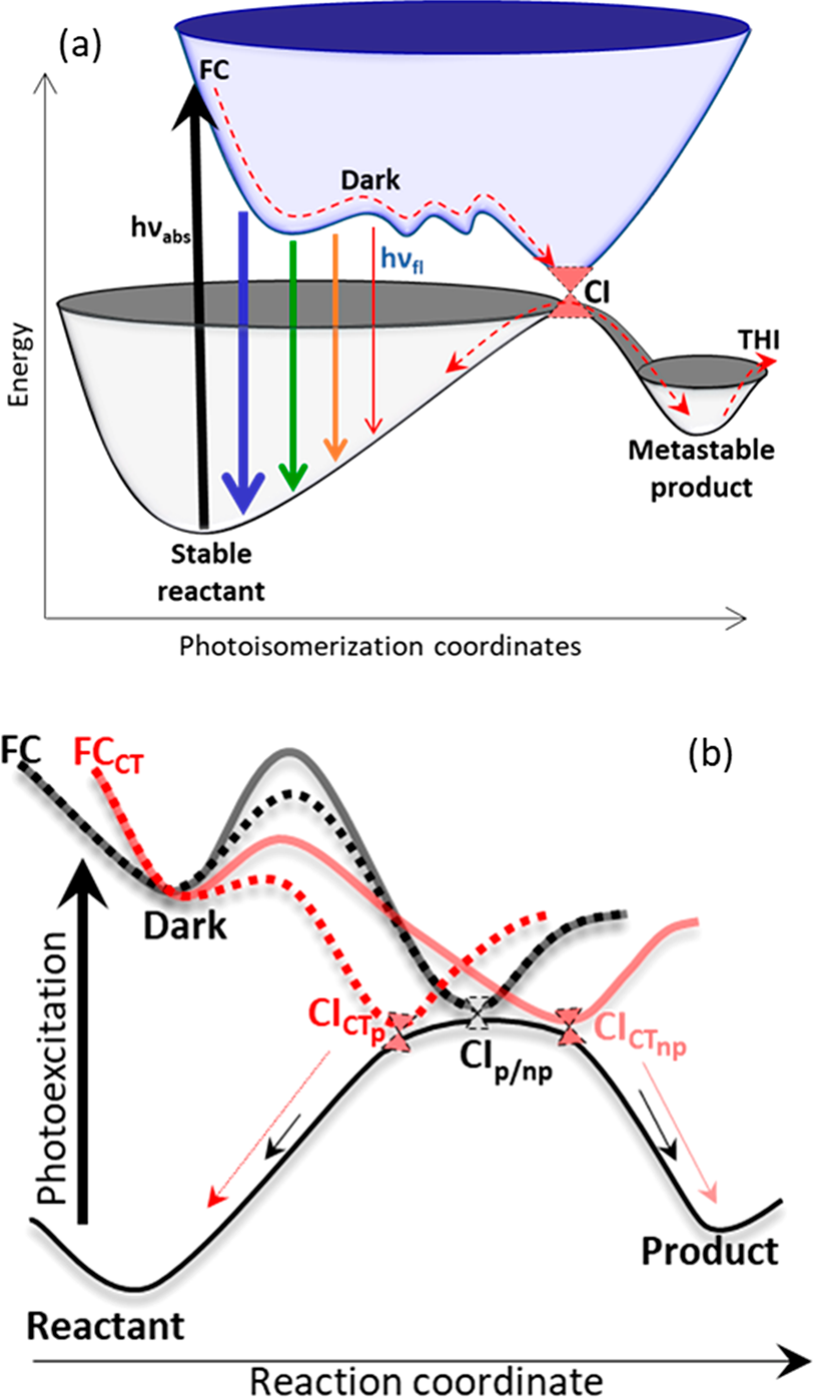
(a) General mechanism for PMM excited state dynamics. Initial excitation of a stable ground state populates the FC states on a repulsive part of the S_1_ PES. This state decays rapidly to a dark state. As the structure evolves along the multidimensional reaction coordinate, the S_1_ → S_0_ transition moment decreases and the spectrum red shifts. The dark state decays to the ground state through a CI, which may be accessed by multiple pathways, which leads to nonsingle exponential dark state decay. From the CI the molecule may relax back to its original isomer or go on to the metastable isomer, which progresses further along the reaction coordinate by THI. (b) Illustration of how substituent and solvent may modify the excited state dynamics and motor efficiency. The initially excited FC state undergoes rapid (<300 fs) decay, which is weakly dependent on the substituent and solvent independent. In contrast the dark state decay is strongly influenced by the substituent and its decay is a function of viscosity, through the solvent displacement by motions along the reaction coordinate and polarity by modification of barrier heights and CI topology. (b) Adapted from ref (70). Copyright 2023 American Chemical Society.

Here we bring together the common features of the excited state dynamics of PMMs and how they respond to simple substituents and changes in their environment. Figure 12a plots ground and excited state PESs as a function of the reaction coordinate, where the reaction coordinate is certainly multidimensional, involving at least torsion and pyramidalization at the C=C axle but also other intramolecular modes and solvent orientation. In all cases there is a well-defined stable ground state conformation of the motor. Torsion about the C=C bond in the ground state leads over a high barrier (as expected for twisting a double bond) to a new minimum associated with a metastable ground state isomer. This pathway is inaccessible at ambient temperatures. All the data described here concern photoexcitation of the stable ground state. Studies of metastable states are few but point to quantitative rather than qualitative differences.^41,61^

Electronic excitation of an allowed ππ* transition localized on the C=C bond initially excites an FC state which fluoresces but also undergoes ultrafast decay because of strong steric repulsion. The reaction is thus driven overwhelmingly in one direction down the PES. This process occurs within at most a few hundred femtoseconds, after which a dark state is populated. Coherent oscillations are detected in FlUC and TA and indicate excitation of vibrational modes in the FC state by the coherent ultrafast optical pulse or in the dark state due to impulsive excitation by FC state decay. That these modes modulate the fluorescence transition moment (non-Condon effect) suggests they arise from oscillations along the reaction coordinate connecting bright and dark states (Figure 12a). There is evidence from the different dark state decay kinetics measured in FlUC and TA that further movement along the reaction coordinate causes the transition dipole moment to decrease further. The dark state decays by accessing the region of a CI with the ground state. The nonsingle exponential decay universally observed suggests multiple pathways to the CI, consistent with a complex landscape for the dark state PES. On accessing the ground state, either the original conformer or the metastable product is populated. Ultimately the yield of the product isomer will depend on the location and topology of the CI, and both can be modified by substituent and solvent.^38,80,81^

Means by which such substituent and solvent effects can modify the reaction coordinate are illustrated in Figure 12b, originally conceived for **1** and its derivatives^70^ but likely applicable to other PMMs. The initial decay of the FC state is dominated by intramolecular forces, the steric repulsion in the excited state. It is largely independent of solvent polarity and viscosity but somewhat dependent on structure (i.e., the generation of the motor studied) and substituent (Figure 8). In contrast, the dark state decay via the CI is dependent on motor structure, substituent, and solvent polarity and viscosity. It is at this point in the reaction coordinate that the experimentalist can intervene to modify PMM efficiency, and the theorist might most usefully focus efforts to predict and control photoisomerization quantum yield. Increasing solvent viscosity alone invariably slows dark state decay, showing that the reaction is in the Kramers high friction regime, as expected. When analyzed in terms of the solvent shear viscosity, the dependence is approximately η^0.5^. Increasing solvent polarity has the effect of reducing the dark state lifetime. This indicates the importance of charge separation along the reaction coordinate, related to the sudden polarization, which plays a prominent role in isomerization reactions of ethylenic bonds. The polar solvent stabilizes the charge separated state, reducing the barrier to the CI (Figure 12b). On its own, a polar solvent acceleration in dark state decay does not control the metastable product yield in **1**(65) (although there appeared to be a significant polarity dependence of yield in **3**(76)). The most effective control parameter appears to be a combination of solvent polarity and substituent. Building in CT character by a push–pull arrangement of donor and acceptor substituents, coupled with solvent polarity effects, leads to qualitative changes in excited state dynamics and significantly enhances/suppresses metastable product formation in nonpolar/polar solvents, respectively. In Figure 12b this is represented with a solvent dependence of the location (or topology) of the CI. To what extent this effect is tunable by different substituents in different locations on the PMM framework is a topic for future study (section 7).

## 6 Other PMMs Driven by Double Bond Isomerization

**Figure 13:**
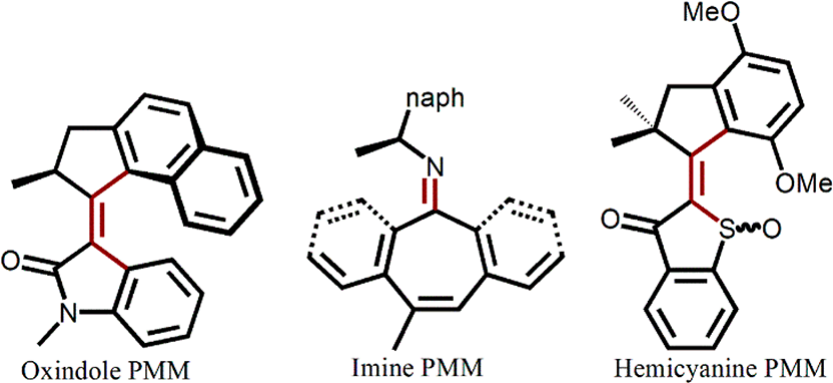
Structures of oxindole, imine, and hemithioindigo motors.

In this section, we mention some other PMMs based on double bond (C=C or C=N) isomerization (Figure 13). Some of these have been designed to address the questions raised above (i.e., the modification of photochemical yield and control of THI), while others have more red-shifted absorption spectra, which may be more suitable for applications in the life sciences. While there are fewer measurements of ultrafast dynamics for these more recently developed systems, the theoretical and steady state data suggest that they represent potentially useful variations on the more established overcrowded alkenes. The coverage here is intended to be illustrative rather than comprehensive.

Feringa and co-workers replaced the three-ring stator with a two-ring oxindole and used NMR and CD spectroscopies to demonstrate unidirectional motor performance, which revealed an isomerization–THI mechanism analogous to **1** and **2**.^82^ The oxindole motors offer advantages in ease of synthesis and functionalization for applications, but they showed a quantum yield of only a few percent. In an effort to tune the efficiency, the rotor was substituted with two electron donating methoxy groups and the mechanism was studied by TA and quantum chemical calculations.^83^ The yield was significantly enhanced. The effect was discussed in terms of calculations, which suggested the charge separation character after C=C rotation was stabilized by the indole ring and more effectively so by the OMe substituents. This stabilization allows the CI region to be reached with less extensive pyramidalization. Further refinements to the structure have been proposed on the basis of calculation.^84^

Greb and Lehn introduced the concept of the chiral imine based PMMs (Figure 13).^85,86^ The mechanism relies on excited state C=N isomerization and a ground state ring inversion involving a nonplanar stator. This yields a four-step mechanism analogous to **1**. Several derivatives were prepared, which allowed a degree of tuning of the inversion rate. The excited state dynamics seem not to have been studied yet, but there are calculations of potential surfaces and excited state dynamics.^87,88^ These suggest the involvement of pyramidalization (or N out-of-plane motion) as well as C=N torsion in the excited state. Interestingly this leads to two possible CIs, one of which favors forward isomer formation while the other repopulates the ground state.^87^ These data thus suggest that substituents favoring one or other CI might significantly modify imine PMM efficiency.

Dube and co-workers introduced a C=C isomerizing PMM with a hemithioindigo structure (Figure 13), which has the desirable property of being activated in the visible region, up to 500 nm, and with kilohertz rotation rates.^89^ Again, the pathway seems to be excited state isomerization followed by THI, as for **1**. Initially not all intermediates were observed, but an elegant synthetic approach was introduced to isolate intermediates and prove directional rotation.^90^ The excited state dynamics were followed by TA, complemented by quantum chemical calculations.^91^ Initial excitation is to an nπ*state, which nevertheless gives rise to a downhill torsional motion in the C=C bond, leading to a new partially twisted structure on the S_1_ surface. From there the system proceeds over a barrier to access the region of a CI with the ground state. A complex landscape with at least one other excited state minimum was detected. A nonproductive triplet pathway was accessible, probably because of the low-lying nπ* states.^92^ Quantum chemical calculations suggest heimthioindigo motors may be capable of very high rotation rates.^93^ Some substituent studies were made using methoxy or alkyl groups. Both electronic and steric factors were found to influence excited and ground state dynamics.

Although beyond the present scope, it is important to note the appearance of several potential molecular motors designed through quantum chemical and molecular dynamics calculations.^69,93,94^ Building on that preliminary design work a biomimetic motor was proposed, with the initial structure of the photochemical unit based on the chromophore of the green fluorescent protein.^95^ One such example suggests the possibility of a two-step motor, retaining the directional excited state reaction in a chiral system but with the barrier to the usually rate-determining THI engineered to be negligible. Then fast unidirectional rotation may be driven by two successive photon absorption and isomerization steps: a two (or half?) stroke motor.^96^ Even an achiral motor system based on retinal has been proposed.^97^ In these examples where there is no separate metastable state, careful control of the PES structure will be required to ensure the fidelity of unidirectional motion.

## 7 Future Work

A fundamental mechanism of PMM excited state dynamics based on C=C isomerization has been presented, and the significant effect of substituent and solvent has been elucidated. The experimental observations have been supported (and sometimes predicted) by quantum chemical calculation. This represents significant progress, but there is more to be done. While excited state dynamics of the various generations of “Feringa motors” have been well characterized, there is space for similar work on the other families. Even for the best studied motors only a few substituents have been investigated, so the exploitation of CT state tuning is in its infancy. Calculations are already helpful in this endeavor and are improving at such a rapid rate that one can envision quantitative prediction of motor efficiency for even quite large PMMs. As the quality of excited state calculations improves, it will become possible to simulate vibrational spectra of transient states, which will provide a further level of detail. However, inclusion of effects of solvent dynamics, which are known to be important, might be challenging, although some work is already appearing.^63,96^

More challenges to synthesis, theory, and spectroscopy are expected to arise as existing PMMs are adapted for applications. Along with optimized efficiency there is an increasing demand for PMMs that operate with red or even near IR excitation (for biomedical applications for example).^98,99^ This may require substantial synthetic modification, which will in turn modulate PMM efficiency. Similarly, there are many potential applications for motors that operate cooperatively, of which **3** is only the simplest example. This motor cooperativity may require motor assemblies with components which operate at different wavelengths. Realization and characterization of such PMMs will provide synthetic, measurement, and calculational challenges for generations to come.

## 8 Methods

The stilbene motif is at the core of overcrowded alkene **1**. Stilbene itself exhibits ultrafast photochemical dynamics;^23,24^ hence the study of PMM excited state dynamics requires, as has been shown, application of the methods of ultrafast spectroscopy. Three methods were applied, and their merits and shortcomings are outlined here.

### 8.1 Femtosecond Transient Absorption (TA)

The workhorse of ultrafast photochemistry is TA, in which an ultrafast pump pulse initiates an excited state reaction, which is then probed by measuring the transmission spectrum of a spectrally broad ultrafast probe pulse as a function of the pump–probe delay time.^100,101^ Typically, the probe spans visible to near IR wavelengths (a “white light continuum”) which is usually generated by focusing an ultrafast pulse into a sapphire (used here) or CaF_2_ plate. The time resolution may be tens of femtoseconds (although 100 fs was used in the studies described here^50^). Alternatively, in transient infrared spectroscopy (TRIR) an IR probe is generated by difference frequency generation and may be several hundred wavenumbers wide.^102^ In either case, TA data are reported as pump-on minus pump-off difference spectra, and methods to optimize signal-to-noise have been described in detail.^100^ The key advantage is that all states formed or disappearing from the sample as a result of pump pulse excitation will be observed, as transient absorptions or bleaches (respectively, positive and negative changes in optical density, ΔOD). The only requirement is that the state probed has an adequate ΔOD. In addition, TA is sensitive to strongly emissive states, which are observed as stimulated emission gain in the probe (i.e., with negative ΔOD). The principal disadvantage of TA is that electronic spectra are broad and higher excited states are numerous, which may result in crowded and strongly overlapped spectra that are difficult to disentangle. Global analysis has emerged as a helpful tool to tackle this problem.^103−105^

### 8.2 Ultrafast Fluorescence

A key tool for probing PMM dynamics is ultrafast fluorescence spectroscopy. This can be viewed as a pump–probe method where the pump pulse excites the sample and the resulting fluorescence is time resolved by ultrafast sampling with a short probe pulse. Most often sampling is by fluorescence upconversion (FlUC) of a specific emission wavelength in a nonlinear crystal, and the time resolution may be as high as 30 fs.^106−108^ However, recovery of time dependent fluorescence spectra requires laborious reconstruction from FlUC measurements made at multiple emission wavelengths.^107,108^ Alternative broadband upconversion and Kerr gated sampling methods yield spectra directly, offer faster data collection, and give better spectral resolution but have lower time resolution.^109,110^ That the experiment is restricted to measuring fluorescent states is also perhaps its greatest strength. Since any optically allowed state must begin to emit as soon as it is populated, ultrafast FlUC provides an unambiguous observation of relaxation from the Franck–Condon (FC) state free from the overlapping transitions of TA. Further the temporal evolution of the fluorescence spectrum probes exclusively the initial excited state dynamics on the upper surface. Finally, the vibrational coherences often seen in ultrafast spectroscopy are also observed in FlUC, but in that case they can be confidently assigned to modes in the excited electronic state.^48^

### 8.3 Ultrafast Vibrational Spectroscopy

Ultrafast vibrational spectroscopy complements TA by providing finer structural details on the observed transients. Two methods are popular: femtosecond stimulated Raman spectroscopy (FSRS) and TRIR. The latter is an extension of TA to the IR region and thus provides transient IR absorption difference spectra of all species in the system at a given time delay.^102^ The superior spectral resolution in the IR aids assignment, although IR detectors tend to be noisier than those for the visible region and solvent absorption is problematic. The FSRS method is an adaptation of TA in which the probe comprises two pulses: the ultrafast white light continuum overlapped with a temporally broad (ca. 1 ps) spectrally narrow (ca. 10 cm^–1^) “Raman pump” pulse. The probe pulse combination generates an instantaneous stimulated Raman spectrum, which has the spectral resolution of the Raman pulse and the temporal resolution of the continuum, which is then measured as a function of pump–probe delay.^111−114^ The measured signal comprises a number of contributions, which must be carefully measured and separated, and the final spectrum can be complicated by interference with competing signals.^112,115^ These effects are now quite well understood and can be removed or corrected for as described in detail elsewhere, although this complexity is undoubtedly a limitation of the method.^112,115^ An advantage of FSRS is that the Raman pulse can be tuned into resonance with a specific transient absorption and the resonance enhancement used to extract state specific transient Raman spectra.^116,117^ It should be noted that there is a Fourier transform time domain analogue of FSRS, impulsive SRS, which has fewer and simpler background correction problems and can provide outstanding signal-to-noise, but which places high demands on the laser source and experimenter, requiring sub 10 fs pulses, high stability, and long measurement times.^118^
